# A fatal combination of disseminated strongyloidiasis with two bacterial infections in an immunocompromised host

**DOI:** 10.1099/acmi.0.000246

**Published:** 2021-07-22

**Authors:** Vineeta Vini, Sherly Antony, Teena Jacob, Archana Sasimohan, Aneeta Mary Jacob, Mercy John Idikula, Jacob Cherian

**Affiliations:** ^1^​ Department of Microbiology, Pushpagiri Institute of Medical Sciences and Research Centre, Tiruvalla, Kerala, India; ^2^​ Department of Medicine, Pushpagiri Institute of Medical Sciences and Research Centre, Tiruvalla, Kerala, India

**Keywords:** hyperinfection syndrome, *Strongyloides stercoralis*, *Aeromonas sobria*, disseminated strongyloidiasis

## Abstract

**Introduction:**

*Strongyloides stercoralis* is an intestinal nematode that is endemic in tropical countries. It can have a variable presentation ranging from asymptomatic eosinophilia in immunocompetent hosts to disseminated disease with sepsis in immunocompromised hosts.

**Case report:**

We report a case of chronic diarrhoea and decreased appetite in a 53-year-old man. He was a chronic alcoholic with diabetes, hypertension and dyslipidaemia and had earlier been treated for pulmonary tuberculosis. He was treated symptomatically for loose stools at a primary health care facility without relief. Following referral to our tertiary care centre, microscopic examination of the stool showed numerous larvae and a few eggs of *Strongyloides stercoralis*. Additionally, *Aeromonas sobria* was isolated from stool culture. The patient was discharged following improvement with a combination therapy of ivermectin, albendazole and ciprofloxacin. However, within 3 days, he was readmitted and succumbed to *Escherichia coli* sepsis.

**Conclusion:**

Strongyloidiasis can be diagnosed easily using a very simple but often neglected investigation, namely stool microscopy. This provides an early diagnosis, based on which prompt treatment with the appropriate antihelminthics can be started, thereby reducing the probability of disseminated infection. Disseminated strongyloidiasis is a medical emergency with a poor prognosis, especially in an immunocompromised state. Such patients should be treated aggressively with antihelminthics. They must be monitored for sufficient duration in the hospital for early signs of complication. Their discharge from hospital should be planned based on a negative stool microscopy report in addition to clinical improvement, so as to decrease the mortality reported for both untreated and treated individuals.

## Introduction

*Strongyloides stercoralis* is a geo-helminth, which causes strongyloidiasis, a neglected tropical disease [1, 2]. It was first reported in 1876 in French soldiers with severe diarrhoea while returning from Vietnam [3, 4]. It affects nearly 370 million people worldwide with a prevalence of >60 % in endemic areas such as Southeast Asia, Sub-Saharan Africa, the West Indies and Latin America [3]. The prevalence of this disease in India is ~6.6 % in the community and ~11.2 % in hospital settings [4].

The larvae thrive in warm, moist, or wet soil. Hence, walking barefoot and engaging in work involving skin contact with soil in the presence of low sanitary standards are the major risk factors, with the mode of infection being transcutaneous. In immunocompetent individuals, the parasitism remains quiescent. However, in immunocompromised individuals, the helminth multiplies, leading to hyperinfection syndrome.

Defective cell-mediated immunity is the major contributing factor for the spread of larvae from the intestinal tract, leading to hyperinfection syndrome and dissemination. The predisposing factors for developing hyperinfection syndrome are living in an endemic region, corticosteroid therapy, infections with human T-lymphotropic virus-1 (HTLV-1), chronic malnutrition, malignancies, organ transplantation, diabetes mellitus, chronic obstructive pulmonary disease (COPD), chronic alcoholism, chronic renal failure and feeding on the breast milk of an infected mother [5]. However, this association between immunosuppression and hyperinfection/disseminated strongyloidiasis does not apply to HIV-AIDS [6]. Hyperinfection syndrome in association with corticosteroid therapy is particularly noteworthy in that it has been described regardless of dose, duration, or route of administration [7]. Even short courses (6–17 days) of steroids in immunocompetent patients have been associated with hyperinfection syndrome and death [8].

## Case report

A 53-year-old male from Kerala, South India presented with loose stools, which were watery in nature and without blood, three–four episodes per day for 3 weeks. He complained of vague abdominal pain on and off, decreased appetite and weight loss of 3–4 kg over 1 month. He gave no history of associated fever, vomiting, urticarial rashes, or any respiratory symptoms.

In addition to having alcohol use disorder, he was a known case of type II diabetes mellitus, hypertension and hyperlipidaemia and was on regular oral medications for the previous 2 years. He also experienced recurrent episodes of stroke, which resulted in a left-sided hemiparesis 2 years previously. He was also diagnosed as having pulmonary tuberculosis (sputum-positive), for which he had been on anti-tubercular therapy (ATT) for the previous 6 months. He denied intravenous (IV) drug abuse or any high-risk sexual behaviour. He was not on steroids or immunosuppressive drugs and gave no history of travel outside the state. He reported consumption of only cooked food and there was no food intolerance. All his family members were reported to be healthy.

He was a cattle farmer and worked barefoot on his cattle farm; he also had a shallow pond near to his home where he used to grow freshwater fish for consumption. To his knowledge there was no practice of open field human defecation in the farm area.

He was treated symptomatically at primary health care facilities and since there was no relief, he was referred to this tertiary care centre.

On clinical examination, he was not febrile and vitals were within normal limits. He had bilateral pitting pedal oedema and macular erythematous rashes over the anterior aspect of both the legs for 2 weeks, which were non-migratory in nature. Systemic examination was normal.

### Investigations

Routine blood investigations showed decreased haemoglobin (Hb) 9.6 %, elevated total count (TC) 13 600 cells cumm^−1^ with differential count (DC), polymorphs 90 %, lymphocyte 6 %, eosinophil 2 % and monocytes 2 %, an erythrocyte sedimentation rate (ESR) of 78 mm h^−1^, and serum electrolytes showed hypokalaemia (potassium – 2.6 meq l^−1^). His random blood sugar on admission was elevated (274 mg dl^−1^). He tested negative for viral markers of human immunodeficiency virus (HIV), hepatitis C virus (HCV) and hepatitis B virus (HBV). Ultrasonography (USG) of the abdomen showed fatty hepatomegaly with focal lesions in the liver, for which computed tomography (CT) of the abdomen and pelvis with contrast was performed. This showed several hypodense lesions involving segment 5 of the liver with mild hepatomegaly. CT of the lungs showed cavitation in the upper lobes of both lungs. Upper gastrointestinal endoscopy was performed, which was normal. Stool was sent for microscopy and bacteriological culture.

**Fig. 1.**
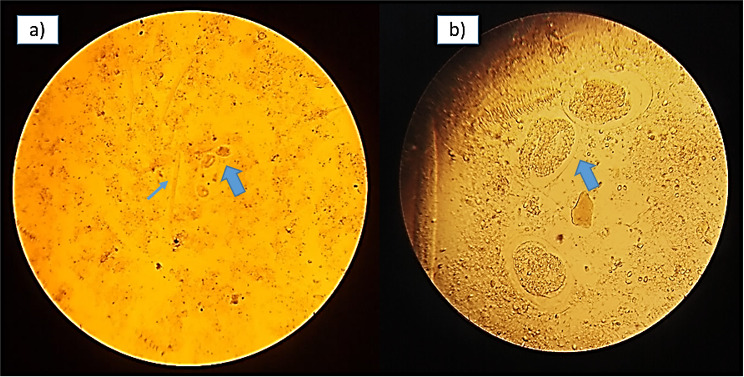
**(a**) Stool microscopy showing rhabditiform larvae (blue thin arrow) and eggs (blue thick arrow) of *S. stercoralis*. Image taken at 100× magnification. (b) Enlarged view of the eggs (blue thick arrow) of *S. stercoralis* (rarely found in stool microscopy). Image taken at 400× magnification.

**Fig. 2.**
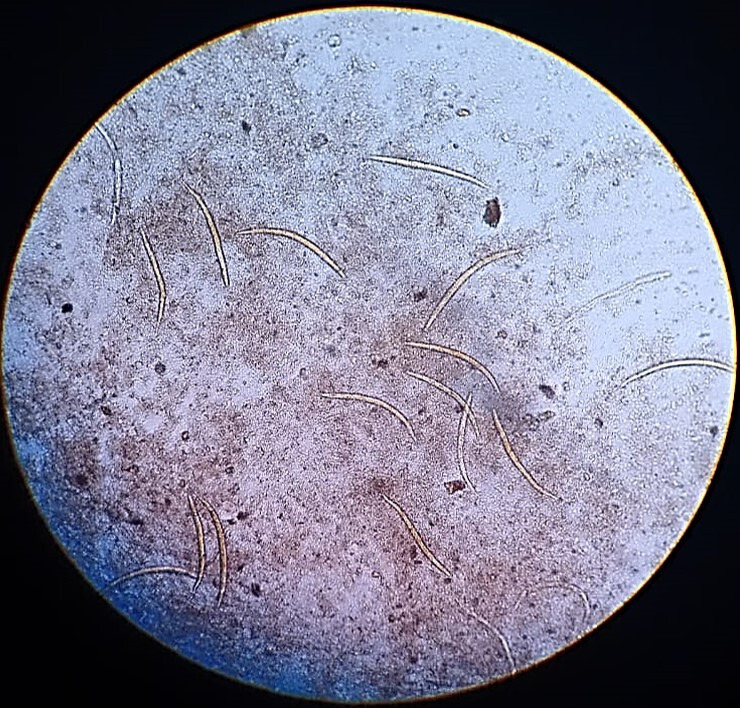
Stool microscopy showing rhabditiform larvae of *S. stercoralis*, which were motile at the time of stool collection. Image taken at 100× magnification.

Macroscopically, the stool was watery, yellowish coloured, with no mucus or blood. Wet preparation revealed numerous motile larvae in the rhabditiform stage, which measured 100–400 µm in length×15–20 µm in width and had a short buccal cavity. There were a few oval shaped eggs, ~50–60×30–35 µm in size, with larvae, suggestive of *S. stercoralis*. There were no pus cells or red blood cells. (Fig. 1) Stool microscopy following the formal ether concentration technique did not show any additional findings. Stool microscopy was repeated with freshly collected stool samples on three separate occasions, which also demonstrated motile rhabditiform larvae of *S. stercoralis* (Fig. 2). In addition, agar culture method was performed to visualize the positive trail sign. Nearly 2 g of freshly passed stool was placed on an agar plate. The plate was sealed with tape and then incubated at room temperature for 2 days. The plate was later examined and showed a ‘positive trail sign’, that is, there were tracks present as the bacteria were carried over the agar by the migrating larvae. The agar culture method was to be repeated after therapy, if microscopy of stool did not reveal larvae, as it is a more sensitive test for assessing the elimination of the parasite.

The bacteriological culture of stool yielded *Aeromonas sobria,* which was sensitive to ciprofloxacin, chloramphenicol, tetracycline and cotrimoxazole. Stool occult blood (Hemospot kit, standard guaiac method, Tulip Diagnostics, Goa, India) and *Clostridium difficile* toxin (VIDAS difficile toxin A and B, bioMérieux SA) were negative.

### Diagnosis

Hyperinfection syndrome with disseminated strongyloidiasis, alcoholic liver disease, type 2 diabetes mellitus, hypertension, dyslipidaemia and pulmonary tuberculosis.

### Therapy

Therapy included blood sugar and electrolyte correction and the patient was empirically started on ciprofloxacin intravenously (IV) along with parenteral fluids and other supportive measures.

Following the stool microscopy report, a stat oral dose of a combination of albendazole 400 mg and ivermectin 6 mg, followed by once daily for 2 days, was given. The patient was continued on ciprofloxacin IV for 4 days. He showed symptomatic improvement by the second day of treatment, his stool became semisolid in consistency and the number of motile larvae in fresh stool showed an appreciable reduction. He was discharged after 4 days on mixtard insulin and oral ciprofloxacin 500 mg twice daily for 3 days.

### Outcome and follow up

The patient was readmitted on the third day of discharge with complaints of fever and recurrence of diarrhoea. His blood sugar value at the time of readmission was again elevated (210 mg dl^−1^). His total leucocyte count (TLC) was 12 000 cumm^−1^, with polymorphs 60 %. His blood was sent for culture and empirical therapy was initiated with injected piperacillin/tazobactam and mixtard insulin. Unfortunately, on day 2 of readmission he expired. *Escherichia coli* was isolated from his blood culture 3 days after his readmission, sensitive to cephalosporins, carbapenems, piperacillin/tazobactam and aminoglycosides, but resistant to ciprofloxacin, which he was discharged on for the *Aeromonas* infection.

## Discussion

*S. stercoralis* is an intestinal nematode which is endemic in the rural parts of tropical and subtropical areas. In India it is more common in the southern states in patients with predisposing factors such as impaired bowel movement, chronic steroid use, malignancy, diabetes mellitus and pulmonary tuberculosis [9].

Our patient was a daily wage earner of low socioeconomic status, with multiple associated conditions, namely chronic alcoholism, uncontrolled type II diabetes mellitus and pulmonary tuberculosis, which could have contributed to his waning cell-mediated immune response. In fact, there is a five times higher risk of strongyloidiasis in chronic alcoholics, with or without cirrhosis [10]. The above-mentioned predisposing factors, along with walking bare feet, may have led to the chronic *S. stercoralis* infection in this patient. Although there was no practice of using the fields for open human defecation, the soil is likely to be contaminated with stray or free-roaming pet dog faeces. Humans and dogs are two major hosts for this parasite [11] and it has been identified in humans and domestic dogs in the same rural community in north-east Thailand and in other countries [12]. Another host to be considered as a potential reservoir is cats, in which up to four species with *S. stercoralis* have been detected.

Eosinophils exceeding 5 % on the differential count is considered elevated in most laboratories. A mild to moderate eosinophilia should require ruling out associated tissue helminthic infections. Strongyloides infection is one of the common causes, in addition to trichinella, ascaris, hookworm and visceral larva migrans. However, this patient had a normal peripheral blood eosinophil count. It is reported that at least 25 % of patients infected with *Strongyloides* may not have eosinophilia [13] and that absence of eosinophilia may be associated with a poor prognosis [14]. Loss of weight and appetite, as was seen in our patient, may have been associated with chronic immunosuppression [15]. Fatal hypokalaemia has been reported due to *S. stercoralis* infestation [16], but in this case the hypokalaemia was corrected and could not have contributed to the fatality.

The accompanying coinfection with *A. sobria* in stool may have been from the pond where he cultivated the fish. There is a close association of the *Aeromonas* species with aquatic ecosystems. A similar situation of coinfection with *Aeromonas* species has been reported in a 75-year-old man in Puerto Rico with multiple myeloma [17]. The most common presentation for *Aeromonas* gastroenteritis is a secretory (watery) enteritis, and this may have contributed to or exacerbated the enteric manifestation in this patient [18].

The occurrence of *E. coli* sepsis, which the patient succumbed to, is similar to a previous case report in which the patient succumbed to *E. coli* sepsis after 14 days of hospitalization [19]. The passage of enteric flora (*E. coli*) through disrupted mucosa leading to the bacteraemia suggests disseminated strongyloidiasis [20, 21]. Hence, in this patient the bacteraemia caused by *E. coli* and the USG and CT abdomen findings of lesions involving the liver are strongly suggestive of disseminated strongyloidiasis infection along with translocation of gut bacteria.

As per the Centers for Disease Control and Prevention (CDC) guidelines, management of acute strongyloidiasis includes ivermectin, in a single dose, 200 µg kg^−1^ orally for 1–2 days. Alternatives include albendazole, 400 mg orally two times a day for 7 days [22]. However, both albendazole and thiabendazole are slightly less effective when compared to ivermectin for acute strongyloidiasis [13]. For disseminated infection, ivermectin 200 µg kg^−1^ per day needs to be continued orally until negative stool examination persists for 2 weeks [7]. In patients with *Strongyloides* detected on stool examination and persistent symptoms, follow-up stool examnations should be performed 2–4 weeks after treatment to confirm clearance of infection. If recrudescence of larvae is observed, retreatment is indicated.

This patient was treated with a combination of ivermectin and albendazole, as has been suggested in certain case reports of the improved efficacy of combination treatment with both [23]. However, death could not be prevented in this case, as the mortality in disseminated infection is very high even when appropriate therapy is instituted. The mortality rate among disseminated strongyloidiasis patients is almost 100 % if untreated and exceeds 40–70 % in treated individuals [21]. In addition, a strong association of untreated or improperly controlled diabetes with strongyloidiasis treatment failure is documented [24].

During the patient’s previous visits to the peripheral health care facility with complaints of diarrhoea, he had been treated only symptomatically, without any investigation, in spite of a lack of improvement in his condition. A stool sample for microscopic examination, which, if it had been performed, would have provided an early diagnosis and enabled the initiation of antihelminthic treatment, which may have benefited the patient.

## Conclusion

This case demonstrates the importance of stool microscopy, a simple yet often overlooked or frivolously conducted test. Microscopic examination of the stool sample was sufficient for an early specific therapeutic intervention in this case.

Hyperinfection syndrome should always be considered to be a medical emergency and treatment should be initiated without delay. Dissemination is often associated with translocation of gut bacteria resulting in sepsis or bacteraemia, which maybe fatal for the patient. Hence, there is a need for aggressive therapy with antihelminthics combined with antibiotics as used for empirical treatment of septicaemia.

The risk factor for disseminated strongyloidiasis in this patient was chronic immunosuppression due to tuberculosis, alcoholic liver disease and type II diabetes mellitus. These predisposing factors should alert the treating physician to the increased probability of progression to a disseminated state. Hence, such patients should only be treated and discharged following a negative stool microscopy report in addition to clinical improvement. This approach will enable monitoring and management of the complications likely to arise in the immediate post-intervention phase.

The association of corticosteroids and exacerbation of *Strongyloides* infection may be particularly born in mind at present, as steroids are an essential component of coronavirus disease 2019 (COVID-19) management.

## References

[1] Requena-Méndez A, Chiodini P, Bisoffi Z, Buonfrate D, Gotuzzo E (2013). The laboratory diagnosis and follow up of strongyloidiasis: a systematic review. PLoS Negl Trop Dis.

[2] Teixeira MC, Pacheco FT, Souza JN, Silva ML, Inês EJ (2016). *Strongyloides stercoralis* infection in alcoholic patients. Biomed Res Int.

[3] Tuyizere A, Ndayambaje A, Walker TD, Bayingana C, Ntirenganya C (2018). Prevalence of *Strongyloides stercoralis* infection and other soil-transmitted helminths by cross-sectional survey in a rural community in Gisagara District, Southern Province, Rwanda. Trans R Soc Trop Med Hyg.

[4] Schär F, Trostdorf U, Giardina F, Khieu V, Muth S (2013). *Strongyloides stercoralis*: global distribution and risk factors. PLoS Negl Trop Dis.

[5] Montes M, Sawhney C, Barros N (2010). *Strongyloides stercoralis*: there but not seen. Curr Opin Infect Dis.

[6] Viney ME, Brown M, Omoding NE, Bailey JW, Gardner MP (2004). Why does HIV infection not lead to disseminated strongyloidiasis?. J Infect Dis.

[7] Mejia R, Nutman TB (2012). Screening, prevention, and treatment for hyperinfection syndrome and disseminated infections caused by *Strongyloides stercoralis*. Curr Opin Infect Dis.

[8] Ghosh K, Ghosh K (2007). *Strongyloides stercoralis* septicaemia following steroid therapy for eosinophilia: report of three cases. Trans R Soc Trop Med Hyg.

[9] Chordia P, Christopher S, Abraham OC, Muliyil J, Kang G (2011). Risk factors for acquiring *Strongyloides stercoralis* infection among patients attending a tertiary hospital in south India. Indian J Med Microbiol.

[10] de Souza JN, Oliveira CD, Araújo WA, Souza A, Silva ML (2020). *Strongyloides stercoralis* in Alcoholic Patients: Implications of Alcohol Intake in the Frequency of Infection and Parasite Load. Pathogens.

[11] Nagayasu E, Htwe MP, Hortiwakul T, Hino A, Tanaka T (2017). A possible origin population of pathogenic intestinal nematodes, Strongyloides stercoralis, unveiled by molecular phylogeny. Scientific Reports.

[12] Sanpool O, Intapan PM, Rodpai R, Laoraksawong P, Sadaow L (2020). Dogs are reservoir hosts for possible transmission of human strongyloidiasis in Thailand: molecular identification and genetic diversity of causative parasite species. J Helminthol.

[13] Buonfrate D, Requena-Mendez A, Angheben A, Muñoz J, Gobbi F (2013). Severe strongyloidiasis: a systematic review of case reports. BMC Infect Dis.

[14] Igra-Siegman Y, Kapila R, Sen P, Kaminski ZC, Louria DB (1981). Syndrome of hyperinfection with *Strongyloides stercoralis*. Rev Infect Dis.

[15] Ghoshal UC, Ghoshal U, Jain M, Kumar A, Aggarwal R (2002). *Strongyloides stercoralis* infestation associated with septicemia due to intestinal transmural migration of bacteria. J Gastroenterol Hepatol.

[16] Kane MG, Luby JP, Krejs GJ (1984). Intestinal secretion as a cause of hypokalemia and cardiac arrest in a patient with strongyloidiasis. Dig Dis Sci.

[17] Glenn K, Lindholm DA, Meis G, Watts L, Conger N (2017). Case report: a case of recurrent *Strongyloides stercoralis* colitis in a patient with multiple myeloma. Am J Trop Med Hyg.

[18] Janda JM, Abbott SL (2010). The genus *Aeromonas*: taxonomy, pathogenicity, and infection. Clin Microbiol Rev.

[19] Fasih N, Irfan S, Sheikh U, Beg MS (2008). A fatal case of gram negative bacterial sepsis associated with disseminated strongyloidiasis in an immunocompromised patient. J Pak Med Assoc.

[20] Heyworth MF (1996). Parasitic diseases in immunocompromised hosts: Cryptosporidiosis, isosporiasis and strongyloidiasis. Gastroenterol Clin North Am.

[21] Sipali AM, Damidao AOM, Simionato CS (1991). Small bowel bacterial overgrowth in strongyloidiasis. Digestion.

[22] CDC Parasite Strongyloides (2021). Resource for health Professionals. https://www.cdc.gov/parasites/strongyloides/health_professionals/index.html#tx.

[23] Pornsuriyasak P, Niticharoenpong K, Sakapibunnan A (2004). Disseminated strongyloidiasis successfully treated with extended duration ivermectin combined with albendazole: a case report of intractable strongyloidiasis. Southeast Asian J Trop Med Public Health.

[24] Hays R, Giacomin P, Olma L, Esterman A, McDermott R (2017). The relationship between treatment for *Strongyloides stercoralis* infection and type 2 diabetes mellitus in an Australian Aboriginal population: A three-year cohort study. Diabetes Res Clin Pract.

